# Scientists’ political behaviors are not driven by individual-level government benefits

**DOI:** 10.1371/journal.pone.0230961

**Published:** 2020-05-06

**Authors:** Baobao Zhang, Matto Mildenberger

**Affiliations:** 1 Department of Political Science, Yale University, New Haven, CT, United States of America; 2 Department of Political Science, University of California Santa Barbara, Santa Barbara, CA, United States of America; National Institute of Public Finance and Policy, INDIA

## Abstract

Is it appropriate for scientists to engage in political advocacy? Some political critics of scientists argue that scientists have become partisan political actors with self-serving financial agendas. However, most scientists strongly reject this view. While social scientists have explored the effects of science politicization on public trust in science, little empirical work directly examines the drivers of scientists’ interest in and willingness to engage in political advocacy. Using a natural experiment involving the U.S. National Science Foundation Graduate Research Fellowship (NSF-GRF), we causally estimate for the first time whether scientists who have received federal science funding are more likely to engage in both science-related and non-science-related political behaviors. Comparing otherwise similar individuals who received or did not receive NSF support, we find that scientists’ preferences for political advocacy are not shaped by receiving government benefits. Government funding did not impact scientists’ support of the 2017 March for Science nor did it shape the likelihood that scientists donated to either Republican or Democratic political groups. Our results offer empirical evidence that scientists’ political behaviors are not motivated by self-serving financial agendas. They also highlight the limited capacity of even generous government support programs to increase civic participation by their beneficiaries.

## Overview

On Earth Day 2017, thousands of scientists and their supporters protested the Trump administration in Washington, DC as part of the March for Science; marches in other American cities drew tens of thousands [1]. Yet, other scientists express profound misgivings about such efforts to engage in partisan politics. They criticize their colleagues for having “crossed the imaginary line” from researcher to activist, worried that political advocacy might break the social contract underlying public science and research funding [2]. From this perspective, scientists’ credibility as impartial actors requires withdrawing from political life [3].

Other scientists defend colleagues’ political engagement [4]. These scientists believe passive information provision is insufficient in a world where objective facts are the subject of political debate [5, 6]. If political actors reject or discount severe risks to the public, it might be unethical for scientists to stay silent for fear that future research funding could be compromised [5].

These debates have spilled out into the public sphere. Many American political actors sharply question scientists’ motives. Some have asserted that scientists’ political activity reflects partisan preferences or self-interest in receiving government funding. For example, in recent decades, the Republican Party has criticized scientists, threatening to cut science funding and ignore evidence-based policies [7]. Similarly, American conservatives report over-time decline in trust in scientists [8]. After the March for Science, the percentage of conservatives who agreed that “scientists care less about solving important problems than their own personal gain” increased [9].

These critiques of scientists echo arguments about policy feedback effects. Researchers who study policy feedback argue that that government programs, such as subsidies for higher education, can affect their beneficiaries’ political attitudes and behaviors [10, 11]. For instance, the GI Bill increased the civic participation of the veterans who fought in WWII, particularly those who came from disadvantaged backgrounds [12]. Likewise, the National Defense Education Act of 1958, the Higher Education Act of 1965, and Title IX of the 1972 Education Amendments expanded higher education opportunities to women and thus increased their political participation [13]. Researchers suggest that government programs that support higher education created engaged citizens through both resource effects (i.e., increasing the recipients’ economic opportunities) and interpretative effects (i.e., making the recipients feel valued by the government) [14]. Could these theories extend to scientists, and thus validate claims that scientists’ political advocacy reflects vested interests in government resource provision?

Yet, despite contentious debates about the role of scientists in politics and policymaking, arguments about scientists’ political motives have rarely been examined empirically. In this paper, we use a natural experiment to test whether a major government subsidy for science education, the National Science Foundation Graduate Research Fellowship (NSF-GRF) Program, increased civic engagement by scientists. Contrary to partisan critiques of scientists’ political behavior, our study finds no robust evidence that scientists’ motivations to engage in science-related and non-science-related political advocacy are a function of individual-level government benefits provision. Scientists who did not benefit from the NSF-GRF, compared with scientists who benefited but are otherwise similar, were no less likely to vote, donate to political candidates, or participate in the March for Science. Consequently, our research provides credible empirical evidence against rhetorical arguments that link scientists’ political behavior with self-interested financial agendas. It also highlights the limited capacity of even generous government support to trigger positive policy feedbacks.

## Materials and methods

### Empirical research strategy

Our empirical strategy leverages a natural experiment that uses the NSF-GRF to examine whether receiving government benefits shapes scientists’ willingness to engage in political advocacy. This U.S. government award provides a three-year tuition scholarship for graduate studies in the nature and social sciences as well as a living stipend for three years. NSF-GRF applications are highly competitive; in recent years, only about one-third of all applicants receive any recognition from the NSF. Fellowship awardees are given tuition scholarships and stipends; honorable mentions are recognized for their achievement but receive no financial benefits.

Our research subjects are a special group of fellowship applicants who received either the award (treated group) or honorable mention (control group) in any given year after receiving honorable mention in the *previous* year. For instance, our research subjects for the year 1998 are fellowship awardees and honorable mention recipients who received honorable mentions in 1997. Conditional on performance in the previous year and other pre-treatment covariates (e.g., application year, field of study, undergraduate university, graduate university at time of application, gender, ethnicity, and immigration status), we argue that whether an applicant receives a fellowship or honorable mention in this subsequent year is conditionally as-if random. Social psychologists have used a similar research design to determine that winning an NSF-GRF increased an individual’s likelihood of completing graduate school but did not improve the individual’s labor market outcome [15].

A total of 6,428 applicants between 1995 and 2016 were eligible for inclusion in our sample; the applicants and their basic biographical information are publicly available online. Given budget constraints, we randomly sampled 2,210 applicants using stratified sampling; respondents within each year/field-of-study stratum had a 1/3 probability of being sampled. Some strata did not have at least one subject in each condition; we eliminated those strata and were left with 2,119 subjects. For each subject, we collected the following information: full name at the time of the NSF application, undergraduate institution, and graduate institution. Using only this information, we were able to locate current contact information (e.g., email addresses, mailing address, web contact form URLs) using public online searches. We could not find any contact information for 27 subjects (1.2% of the sample), and so they were dropped from the study.

Critically, our study focuses on causal inferences regarding the drivers of scientists’ mobilization, not descriptive inferences about scientists’ political advocacy. Correspondingly, we choose a sampling strategy tailored to causal inference-making over a design that optimizes a descriptively representative sample of all American scientists. While our subjects are not a random sample of American scientists, they represent a wide range of disciplines (e.g., computer scientists, engineers, biologists) and research institutions (e.g., universities, private companies, the government).

### Data

We conducted an original survey of individuals who applied for the NSF-GRF during graduate school between 1995 and 2016 in late April 2017, in the immediate aftermath of the March for Science. To recruit individuals, we emailed a link to the Qualtrics-hosted survey to scientists in our sample who have an email address we can locate (*N* = 2, 090). We contacted a subset of individuals without email addresses through web contact forms (*N* = 4) or via postal mail invitations (*N* = 25). We recontacted respondents who did not take the survey a week later reminding them to take the survey. We received complete responses from 499 individuals or 23% of our sample; our analysis used responses from 408 subjects because we selected year/field-of-study blocks with at least one winner and one non-winner.

We report summary statistics associated with our sample in S1 Data of S5, S6, S7 and S8 Tables. In particular, we find no significant differences between survey respondents and non-respondents’ background characteristics besides that respondents are more likely to be male and have applied for the fellowship in later years.

Our survey measured a series of political attitudes and behaviors, including 1) the subjects’ support for the March for Science, 2) their attitudes towards government funding for science, 3) their public communication of research findings, 4) their political identity, and 5) their willingness to donate to non-profit organizations (including ones with political agenda) through a donation experiment, detailed in the S1 Data of S1 File. There, we also provide a full copy of our survey instrument.

For all individuals in our sample (irrespective of whether an individual responded to our survey), we also compiled applicants’ political donation records using the OpenSecrets.org’s Donor Lookup tool. We manually matched applicants to the political donation records via name, city, and employer (if available). Out of 2,119 subjects, we were able to match 114 subjects (5.4%) to political donations; we coded unmatched subjects as not having made any political donations. We detail the procedure used to match subjects with donation records in the S1 Data of S1 File.

### Balance tests

Our research design depends on the presence of balance in subjects’ background characteristics between our treated and control groups. To evaluate this feature of our design, we analyzed the natural experiment as a block-randomized experiment (assuming complete randomization within block), where each block is a year/field-of-study. There are a total of 56 year/field-of-study blocks. For each background characteristic, we used the following inverse propensity-weighted (IPW) linear regression to estimate the differences in applicant characteristics between the award winners and non-winners:

$$\begin{array}{c} WSS\left(\beta ,p\right)=\sum_{i=1}^{N}\frac{1}{p_{i}}{\left[X_{i}-(\alpha +\beta A_{i})\right]}^{2} \end{array}$$ (1)

where *p*_*i*_ is the probability that subject *i* is in the condition that they are in given their year/field-of-study, *X*_*i*_ is the background characteristic of subject *i*, and *A*_*i*_ is an indicator variable for subject *i*’s award status. $\hat{\beta }$ is the estimate of the difference in the background characteristic between the winners and non-winners. We used IPW linear regression because subjects have different probability of getting an award depending on their year/field-of-study. These probabilities range from 0.17 to 0.83, with a mean of 0.45 and a median of 0.50.

We performed balance tests to assess whether the award winners are different from the honorable mentions along five metrics: 1) undergraduate university is an Ivy League, 2) undergraduate university is an Ivy League Plus (the Ivy League plus MIT, Caltech, Stanford, University of Chicago, Duke, and UC Berkeley), 3) graduate university (at the time of application) is an Ivy League, 4) graduate university (at the time of application) is an Ivy League Plus, 5) predicted gender of the applicant. The gender of the applicant was predicted using first name data from the U.S. Social Security Administration (SSA). We performed web searches for those applicants with gender-neutral names or names not within the SSA database to determine their gender.

**Fig 1:**
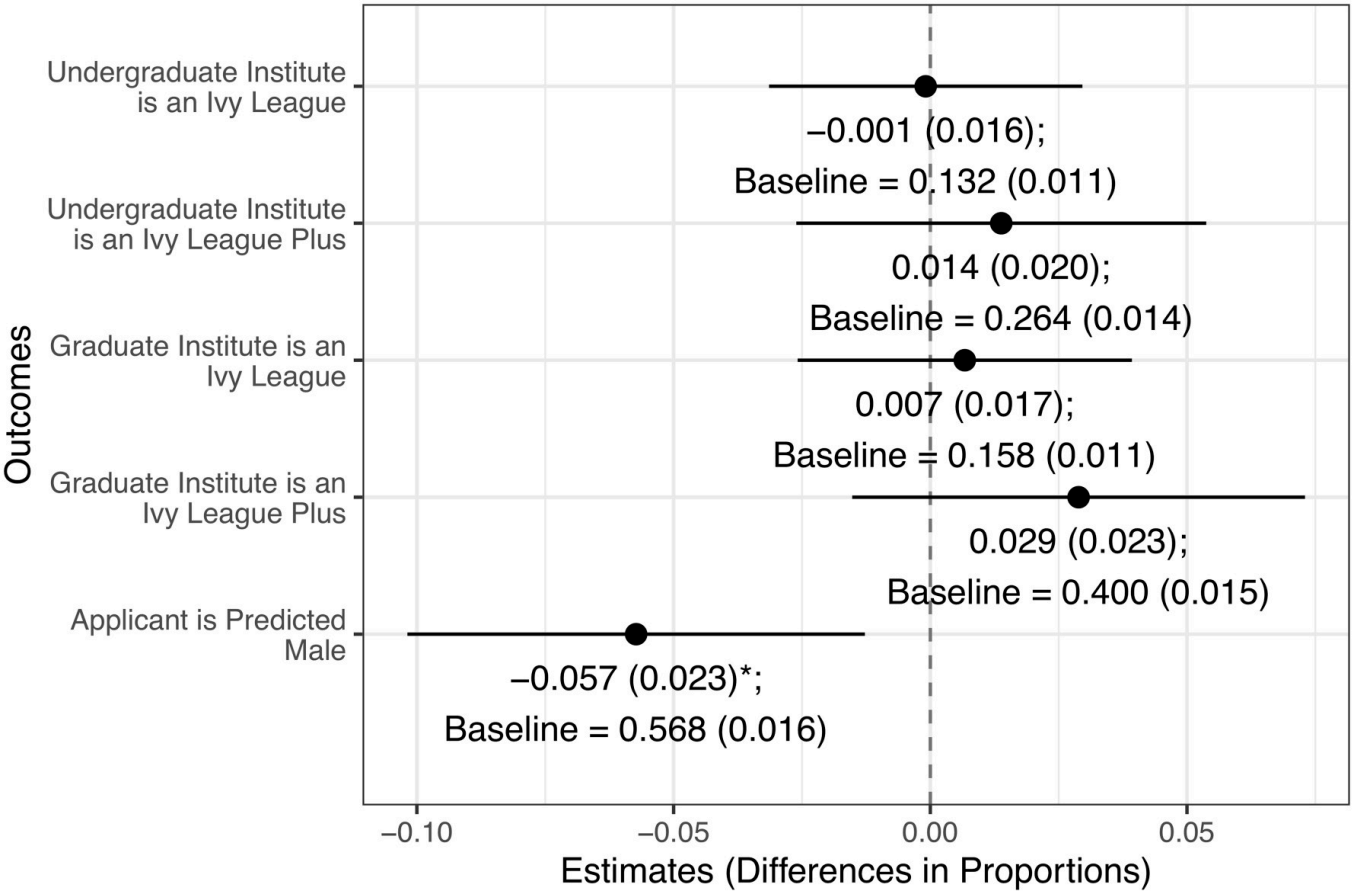
Comparing differences in background characteristics between winners and non-winners. *N* = 2, 119. We find a slight imbalance between treated and control groups only in predicted gender; however, the groups are balanced on the quality of undergraduate university and quality of graduate university.

In Fig 1, we find that award winners and non-winners are not statistically different when it comes to the quality of their undergraduate or graduate institutions, though winners are 5.7 percentage points (SE = 2.3) less likely to be predicted male compared with non-winners. This difference is perhaps not surprising because the NSF seeks to increase diversity in science education; as a result, application reviewers might favor women and ethnic minorities. To address this imbalance in background covariates, we conditioned on predicted gender (along with self-reported racial identity and place of birth in the survey analysis), along with the quality of education background covariates, in subsequent analyses.

Aside from gender, we find no statistical or substantive differences between in survey sample winners and sample non-winners in background covariates (e.g., quality of education, ethnicity, or U.S.-born). Furthermore, these background characteristics do not individually or jointly predict who won a fellowship (see S1 Data of S7 and S8 Tables. These results increase our confidence that treatment assignment (whether an individual received an NSF or honorable mention) is conditionally as-if random in our survey sample.

**Fig 2:**
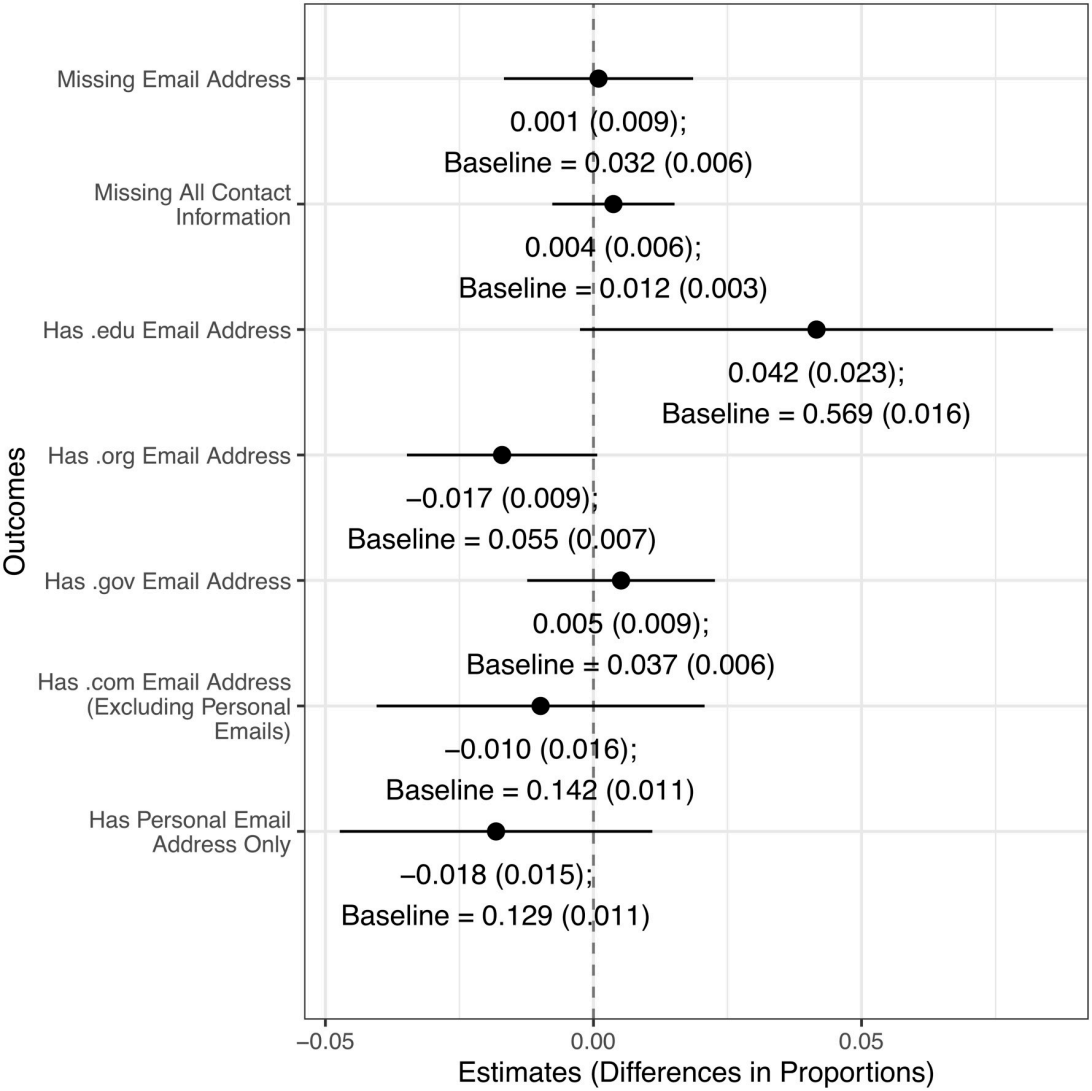
Comparing differences in contact information between winners and non-winners.

Finally, as shown in Fig 2, there are no statistical or substantive differences between award winners and non-winners regarding missing email addresses or all other contact information. Those who were given an award were more slightly likely to have a “.edu” email address, suggesting that winners are more likely to work in academia.

### Analysis of main outcomes

We use the following IPW linear regression to estimate the effect of winning a fellowship on the outcome of interest:

$$\begin{array}{c} WSS\left(\beta ,\theta ,\tau ,p\right)=\sum_{i=1}^{N}\frac{1}{p_{i}}{\left[Y_{i}-(\alpha +\beta A_{i}+\sum_{k=1}^{K}\theta_{k}{\bar{X}}_{i,k}+\sum_{k=1}^{K}\tau_{k}A_{i}{\bar{X}}_{i,k})\right]}^{2}, \end{array}$$ (2)

where *Y*_*i*_ is the outcome measure for applicant *i* and all other variables are the same ones described previously. $\hat{\beta }$ is the estimate of the individual-level average treatment effect of receiving a fellowship on the outcome. We mean-centered the background covariates, *X*_*k*_, so that $\hat{\alpha }$ represents the estimate of the mean outcome for the non-winner group (i.e., the baseline). For the donation outcomes, the background covariates include quality of the subjects’ undergraduate and graduate institutions and their predicted gender. For the survey outcomes, the background covariates include those in addition to the subjects’ self-reported racial identity (i.e., as Black/Hispanic/Native American; as Asian) and whether they self-reported as being born in the U.S. We adjusted for the background covariates, even when there is balance in background covariates between winners and non-winners, to increase the precision of our estimates [16].

## Results

**Fig 3:**
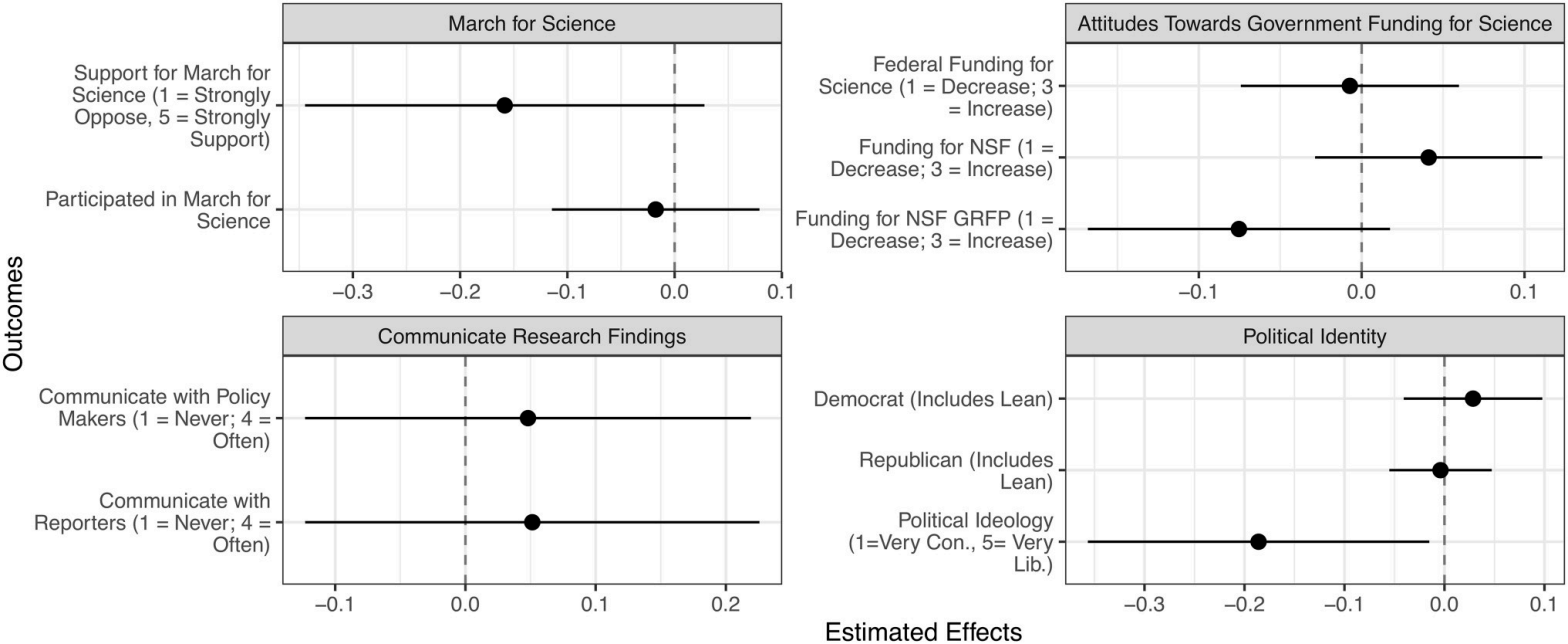
Effect of being awarded the NSF graduate research fellowship on political attitudes and behavior. *N* = 408. The error bars represent 95% confidence intervals calculated from the heteroscedasticity-consistent standard errors. S1 Data of S1 Table contains the effect estimates, standard errors, two-sided *p*-values, and baseline estimates.

Receiving a fellowship did little to change scientists’ political attitudes or self-reported behavior (see Fig 3). Interestingly, winners’ support for the March for Science is 3.5% lower than non-winners’ support; however, this difference is not statistically significant at the 5% level. Award winners do not indicate greater support for federal funding for the sciences, for the NSF, or for the NSF-GRF Program. Neither does winning the fellowship induce respondents to communicate their research findings with policymakers and journalists more frequently. Winning the fellowship do little to alter the respondents’ political party affiliation. Winners are somewhat more conservative in terms of political ideology, but this effect is not robust to an alternative model specification where we do not include background covariates (see S1 Data of S2 Table). Finally, in the donation experiment, respondents’ willingness to donate, whether in general or to organizations with a political agenda, is unaffected by their award status.

**Fig 4:**
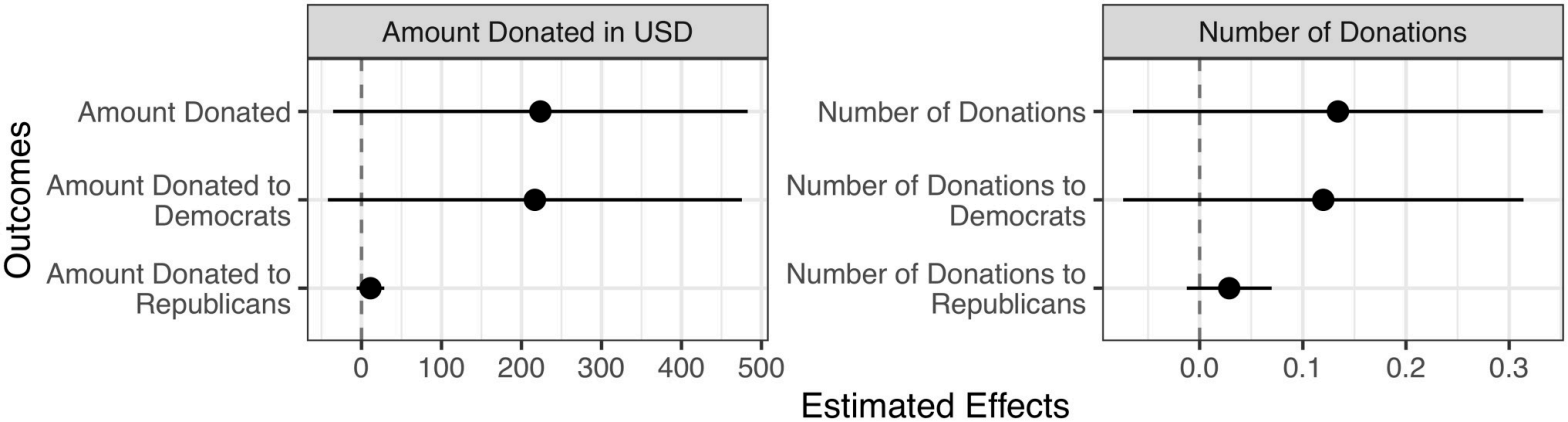
Effect of being awarded the NSF graduate research fellowship on political donations. *N* = 2, 119. The error bars represent 95% confidence intervals calculated from the heteroscedasticity-consistent standard errors. S1 Data of S3 Table contains the effect estimates, standard errors, two-sided *p*-values, and baseline estimates.

We then directly evaluated individuals’ political participation using campaign donation data. This analysis was conducted across all individuals in our sampling frame (*N* = 2, 119), rather than simply across individuals who responded to the survey (*N* = 499). We do not find that applicants who were awarded the fellowship gave larger political donations to Democratic candidates or causes, as seen in Fig 4.

For a further robustness check on the impact of the fellowship on political donation amounts, for each outcome, we conducted a randomization inference test to test the sharp null hypothesis of no individual treatment effect. For our test statistic, we used the Wilcoxon rank statistic because it is robust to outliers [17]. For each outcome, we performed 1,000 simulations assuming the sharp null and the block randomization in our existing data to get the randomization distribution of the test statistic. Each one-sided *p*-value is the proportion of the randomization distribution that is more extreme than our observed test statistic. We find that the two-sided *p*-values (twice the one-sided *p*-values) are all above 0.90; therefore, we cannot reject the null hypothesis of no individual treatment effect for each outcome.

## Discussion

In sum, the null results of award receipt on campaign donations and survey responses suggest that receiving the award did little to change respondents’ political attitudes or behavior. More broadly, we conclude that individual government benefits do not appear to shape scientists’ willingness to engage in either science-related or non-science-related political advocacy. NSF fellowship winners are no more likely than non-winners to support and participate in the March for Science or make political donations.

In the S1 Data of S1 File, we also report the results of two experiments embedded in our survey. In Experiment 1, we randomly divided the survey sample into three groups. The first group received a politically neutral statement about the 2017 March for Science. The second group received the same message with explicit noting that “many March participants wanted to resist Republican party attacks on science and protest federal science policy’s general direction.” A third group received a message that framed the perceived attacks as coming from the Trump administration rather than the Republican Party. Results are reported in S1 Data of S14 Table. Support for the March for Science is equivalent across all conditions. This suggests our sample’s support for political advocacy is relatively non-responsive to partisan cues.

In Experiment 2, we presented respondents with a simple vignette describing the climate advocacy behavior of a hypothetical scholar. We varied both the scholar’s discipline (atmospheric chemistry vs. economics) as well as the nature of the scholar’s advocacy (communication about atmospheric climate science, public endorsement of a carbon tax, public endorsement of a carbon tax plus endorsement of political candidates who support carbon taxes). Results are reported in S1 Data of S2 Fig. We find that scientists’ comfort with political advocacy does not shift as a function of advocacy content. Scientists are as comfortable with a peer advocating for a carbon tax and endorsing candidates who support climate policies, as they are with a peer who limits their advocacy to climate science narrowly.

These results, taken together with our core political advocacy and donation findings, offer an empirical basis to doubt criticism of scientists as being motivated by self-interested political or financial agendas. Other potential mechanisms might contribute to this null finding. While the size of the benefit is significant (amounting to over $200,000 in recent years) and the program is highly visible (recipients receive regular communication from the NSF), its duration is only three years. Furthermore, the program’s beneficiaries might not view the fellowship as a government social program but rather as just reward for their hard work—much like the many Americans who failed to recognize that Social Security is a government social program [18]. As a result, the recipients might not have acknowledged the need to engage in politics to defend the program or the NSF. Alternatively, scientists may fear the loss of future government funding. The March for Science instructed protesters not to wear attire that would identify their universities, use government-funded websites for advertising the protest, or communicate about the March using their institutional email accounts [19]. Because participating in partisan politics could jeopardize their careers, scientists may be reluctant to speak out.

One likely explanation for these findings is the high baseline levels of political engagement in our sample. Evidence from our survey and other surveys of scientists suggest that scientists, as a whole, are politically engaged; interventions to increase their political engagement is likely to hit a ceiling. As the baseline (non-winner) estimates in S1 Data of S1 and S2 Tables show, non-winners in ours survey sample support increasing government funding for science, endorse the March for Science, and overwhelmingly identify as Democrats. More than one-third of the survey sample participated in the March for Science, a costly form of political participation. Regarding a less costly but common form of civic participation, over 98% of both sample winners and non-winners reported they have registered to vote. We also note that government benefits received at an early stage in an individual’s career may have different long-term effects than benefits received at later stages. Follow-up work could investigate the causal effects of other government grant-making programs.

However, none of these potential explanations provide evidence in support of critics’ key claim: that scientists’ political behavior reflects individual financial self-interest. Even at a moment of heightened science politicization, we cannot find empirical evidence in support of the proposition scientists’ political behavior is shaped by having received individual-level government benefits provision. Scientists and their critics should look elsewhere to make sense of scientists’ political interventions.

## Supporting information

S1 Data Online supplement for: Scientists’ political behaviors are not driven by individual-level government benefits. (PDF)
