# Supplementary material for: Scientists’ political behaviors are not driven by individual-level government benefits
Source: PLoS One. 2020 May 6;15(5):e0230961. doi: 10.1371/journal.pone.0230961 (PMC7202598; doi:10.1371/journal.pone.0230961)
Supplement: S1 Data — (PDF) [file pone.0230961.s001.pdf]

Online Supplement for: Scientists' political behaviors are not driven by individual-level government benefits

Baobao Zhang[1] and Matto Mildenberger[2]

[1]Department of Political Science, Yale University
[2]Department of Political Science, University of California Santa Barbara

March 29, 2020

Repication Materials

Replication materials have been deposited on the Harvard Dataverse, and can be accessed here: `https://dataverse.harvard.edu/dataset.xhtml?persistentId=doi:10.7910/DVN/HVKKHL`

Procedure to Match Subjects with Political Donation Records

For all individuals in our sample (irrespective of whether an individual responded to our survey), we also compiled applicants political donation records using the OpenSecrets.orgs Donor Lookup tool. We manually matched applicants to the political donation records via name, city, and employer (if available). We use the following procedure:

1. Search if any donor matches the subject's first and last name.

2. For female subjects who changed their last name, search using both their maiden names and married names.

3. For subjects with nicknames or more commonly use their middle names, also search using their nicknames or middle names.

4. If there are no name matches, record the subject's donation as $0.

5. If there is a name match, check if the donor's middle name matches the subject's middle name. In addition, check if the donor's employers and locations (i.e., city and state) match the subject's current or past employers or locations. If all three matches, record the matched donor's donation record. If the donor's middle name is not available, download if her employers and location match those of the subject. Out of 2,119 subjects, we were able to match 114 subjects (5.4%) to political donations.

Political donation records were compiled into six outcome variables: 1) amount donated (in USD), 2) amount donated to Democrats or Democratic causes, 3) amount donated to Republicans or Republican causes, 4) number of donations, 5) number of donations to Democrats or Democratic causes, and 6) number of donations to Republicans or Republican causes.

Main Results Tables

The following tables present the numerical results that are used to generate the two figures in the main text.

Table 1: Effect of Being Awarded the NSF Graduate Research Fellowship on Political Attitudes and Behavior ($N = 408$); Including Background Covariates

Outcome	Estimate (SE)	Baseline	p-value
Support for March for Science (1 = Strongly Oppose, 5 = Strongly Support)	-0.158 (0.095)	4.460	0.096
Participated in March for Science	-0.018 (0.049)	0.358	0.721
Federal Funding for Science (1 = Decrease; 3 = Increase)	-0.007 (0.034)	2.921	0.832
Funding for NSF (1 = Decrease; 3 = Increase)	0.041 (0.036)	2.878	0.248
Funding for NSF GRFP (1 = Decrease; 3 = Increase)	-0.075 (0.047)	2.758	0.112
Communicate with Policy Makers (1 = Never; 4 = Often)	0.048 (0.087)	1.617	0.583
Communicate with Reporters (1 = Never; 4 = Often)	0.051 (0.089)	1.689	0.564
Donation Dollar Amount	-0.017 (4.469)	38.852	0.997
Amount Donated to Organizations with Political Agenda (in USD)	1.654 (3.377)	13.180	0.625
Democrat (Includes Lean)	0.029 (0.035)	0.857	0.420
Republican (Includes Lean)	-0.004 (0.026)	0.064	0.880
Political Ideology (1=Very Con., 5= Very Lib.)	-0.186 (0.087)	4.104	0.033

Table 2: Effect of Being Awarded the NSF Graduate Research Fellowship on Political Attitudes and Behavior ($N = 408$); Not Including Background Covariates

Outcome	Estimate (SE)	Baseline	p-value
Support for March for Science (1 = Strongly Oppose, 5 = Strongly Support)	-0.125 (0.100)	4.440	0.212
Participated in March for Science	-0.002 (0.049)	0.351	0.963
Federal Funding for Science (1 = Decrease; 3 = Increase)	0.004 (0.035)	2.916	0.912
Funding for NSF (1 = Decrease; 3 = Increase)	0.044 (0.035)	2.877	0.212
Funding for NSF GRFP (1 = Decrease; 3 = Increase)	-0.071 (0.046)	2.758	0.127
Communicate with Policy Makers (1 = Never; 4 = Often)	0.056 (0.088)	1.612	0.523
Communicate with Reporters (1 = Never; 4 = Often)	0.047 (0.090)	1.690	0.603
Donation Dollar Amount	0.043 (4.458)	38.714	0.992
Amount Donated to Organizations with Political Agenda (in USD)	1.661 (3.340)	13.058	0.619
Democrat (Includes Lean)	0.04 (0.036)	0.851	0.272
Republican (Includes Lean)	-0.013 (0.027)	0.070	0.632
Political Ideology (1=Very Con., 5= Very Lib.)	-0.144 (0.092)	4.077	0.118

Table 3: Effect of Being Awarded the NSF Graduate Research Fellowship on Political Donations ($N = 2,119$); Including Background Covariates

Outcome	Estimate (SE)	Baseline	p-value
Amount Donated	223.63 (132.22)	29.31	0.091
Amount Donated to Democrats	216.76 (132.03)	24.56	0.101
Amount Donated to Republicans	11.22 (8.8)	4.53	0.202
Number of Donations	0.13 (0.1)	0.22	0.186
Number of Donations to Democrats	0.12 (0.1)	0.21	0.226
Number of Donations to Republicans	0.03 (0.02)	0.01	0.172

Table 4: Effect of Being Awarded the NSF Graduate Research Fellowship on Political Donations ($N = 2,119$); Not Including Background Covariates

Outcome	Estimate (SE)	Baseline	p-value
Amount Donated	215.64 (129.85)	29.45	0.097
Amount Donated to Democrats	209.81 (129.68)	24.46	0.106
Amount Donated to Republicans	9.93 (8.35)	4.91	0.235
Number of Donations	0.13 (0.1)	0.22	0.199
Number of Donations to Democrats	0.11 (0.1)	0.2	0.235
Number of Donations to Republicans	0.03 (0.02)	0.01	0.193

Further Details About the Subjects

In this subsection, we present further information about the study subjects.

Table 5: Summary Statistics of Respondents and Non-respondents

	Did Not Take Surve	Took Survey	Difference
Award Winner	0.480	0.511	-0.031 (0.026)
Undergraduate Institute Is an Ivy League	0.134	0.122	0.012 (0.017)
Undergraduate Institute Is an Ivy League Plus	0.271	0.269	0.002 (0.023)
Graduate Institute Is an Ivy League	0.161	0.180	-0.019 (0.019)
Graduate Institute Is an Ivy League Plus	0.419	0.421	-0.002 (0.025)
Applicant is Predicted Male	0.560	0.481	0.080 (0.026)$**$
Year Applied	2007.309	2008.731	-1.422 (0.306)$***$
Field: Chemistry	0.066	0.058	0.008 (0.012)
Field: Computer Science	0.042	0.034	0.008 (0.010)
Field: Engineering	0.183	0.156	0.026 (0.019)
Field: Geosciences	0.023	0.016	0.007 (0.007)
Field: Life Sciences	0.378	0.387	-0.008 (0.025)
Field: Material Sciences	0.001	0.002	-0.001 (0.002)
Field: Math	0.026	0.042	-0.016 (0.010)
Field: Physics and Astronomy	0.064	0.056	0.008 (0.012)
Field: Psychology	0.122	0.132	-0.010 (0.017)
Field: Social Sciences	0.094	0.116	-0.022 (0.016)

Table 6: Predicting Survey Response Using Applicants' Characteristics

Characteristic	Estimate (SE)	p-value
Award Winner	0.016 (0.018)	0.393
Undergraduate Institute Is an Ivy League	-0.025 (0.036)	0.482
Undergraduate Institute Is an Ivy League Plus	0.013 (0.028)	0.641
Graduate Institute Is an Ivy League	0.027 (0.029)	0.361
Graduate Institute Is an Ivy League Plus	-0.006 (0.022)	0.779
Applicant is Male	-0.045 (0.020)	0.021
Year Applied	0.006 (0.001)	$<$0.001
Field: Computer Science	0.003 (0.057)	0.953
Field: Engineering	0.011 (0.041)	0.781
Field: Geosciences	-0.051 (0.065)	0.436
Field: Life Sciences	0.023 (0.039)	0.545
Field: Material Sciences	0.260 (0.520)	0.618
Field: Math	0.136 (0.068)	0.048
Field: Physics and Astronomy	0.016 (0.051)	0.756
Field: Psychology	0.030 (0.045)	0.497
Field: Social Sciences	0.070 (0.047)	0.137
Intercept	-12.496 (3.009)	$<$0.001
N	2119	
F-statistic	F(16, 2102) = 2.453	p-value = 0.001

Table 7: Summary Statistics of the Survey Sample

Characteristic	No Award	Award	Difference
Undergraduate Institute Is an Ivy League	0.123	0.135	0.013 (0.036)
Undergraduate Institute Is an Ivy League Plus	0.245	0.244	$<$-0.001 (0.045)
Graduate Institute Is an Ivy League	0.172	0.187	0.015 (0.040)
Graduate Institute Is an Ivy League Plus	0.387	0.415	0.028 (0.051)
Applicant is Predicted Male	0.509	0.418	-0.092 (0.052)
Self-reported Under-represented Minority (Black, Hispanic, and/or Native American)	0.063	0.074	0.011 (0.026)
Self-reported Asian	0.098	0.095	-0.003 (0.029)
Self-reported Born in the US	0.891	0.888	-0.003 (0.034)
N = 408			

Table 8: Predicting Award Using Survey Sample's Respondent Characteristics

Characteristic	Estimate (SE)	p-value
Undergraduate Institute Is an Ivy League	0.072 (0.111)	0.520
Undergraduate Institute Is an Ivy League Plus	-0.037 (0.086)	0.668
Graduate Institute Is an Ivy League	0.015 (0.084)	0.858
Graduate Institute Is an Ivy League Plus	0.032 (0.068)	0.633
Applicant is Predicted Male	-0.101 (0.053)	0.058
Self-reported Under-represented Minority (Black, Hispanic, and/or Native American)	0.023 (0.106)	0.826
Self-reported Asian	-0.025 (0.089)	0.776
Self-reported Born in the US	0.002 (0.099)	0.984
Intercept	0.532 (0.103)	<0.001
N	408	
F-statistic	F(8, 399) = 0.539	p-value = 0.827

Additional Results

This section includes the results of secondary analyses.

Table 9: Naive Balance Test: Simple Difference-in-Means Between Winners and Non-winners (All Subjects; $N = 2,119$)

Outcome	Estimate (SE)	Baseline	p-value
Undergraduate Institute is an Ivy League	-0.009 (0.015)	0.135	0.560
Undergraduate Institute is an Ivy League Plus	0.001 (0.019)	0.270	0.948
Graduate Institute is an Ivy League	-0.002 (0.016)	0.167	0.897
Graduate Institute is an Ivy League Plus	0.015 (0.021)	0.412	0.476
Applicant is Predicted Male	-0.071 (0.022)	0.576	0.001

Table 10: Effect of Being Awarded the NSF Graduate Research Fellowship on Political Donation Amounts: Randomization Inference Results (Based on 1,000 Simulations)

Outcome	Wilcoxon rank statistic	Two-sided p-value
Amount Donated	526,881.500	0.989
Amount Donated to Democrats	534,369	0.957
Amount Donated to Republicans	512,128.500	0.998

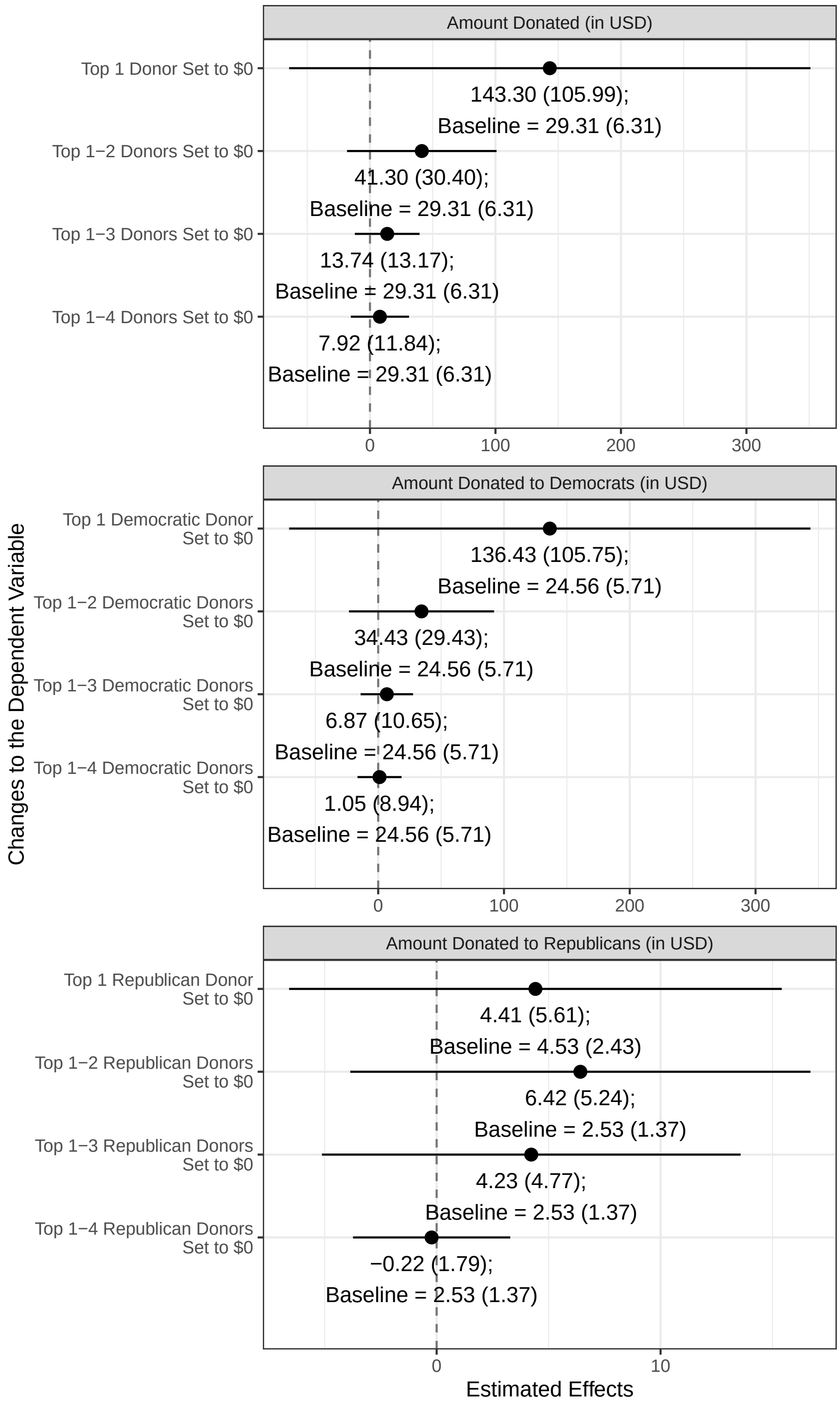

Figure 1: Robustness Check: Effect of Being Awarded the NSF Graduate Research Fellowship on Political Donations ($N = 2,119$) by Setting Top Donations to $0

Table 11: Effect of Being Awarded the NSF Graduate Research Fellowship on Political Donations ($N = 2,119$); Including Only Predicted Gender as A Background Covariate

Outcome	Estimate (SE)	Baseline	p-value
Amount Donated	229.59 (137.23)	29.15	0.094
Amount Donated to Democrats	223.04 (137.1)	24.24	0.104
Amount Donated to Republicans	11.04 (8.75)	4.69	0.207
Number of Donations	0.14 (0.1)	0.22	0.181
Number of Donations to Democrats	0.12 (0.1)	0.2	0.218
Number of Donations to Republicans	0.03 (0.02)	0.01	0.173

Table 12: Interaction Effects of Award and Year of Application on Political Donations ($N = 2,119$)

Outcome	Interaction Effect Estimate (SE)	p-value
Amount Donated	-61.952 (37.572)	0.099
Amount Donated to Democrats	-60.106 (37.564)	0.110
Amount Donated to Republicans	-3.196 (2.492)	0.200
Number of Donations	-0.047 (0.025)	0.064
Number of Donations to Democrats	-0.045 (0.025)	0.071
Number of Donations to Republicans	-0.006 (0.006)	0.346

Table 13: Interaction Effects of Award and Year of Application on Political Attitudes and Behavior ($N = 408$)

Outcome	Interaction Effect Estimate (SE)	p-value
Support for March for Science (1 = Strongly Oppose, 5 = Strongly Support)	-0.013 (0.019)	0.476
Participated in March for Science	-0.005 (0.009)	0.629
Federal Funding for Science (1 = Decrease; 3 = Increase)	0.005 (0.008)	0.557
Funding for NSF (1 = Decrease; 3 = Increase)	0.003 (0.009)	0.725
Funding for NSF GRFP (1 = Decrease; 3 = Increase)	-0.007 (0.01)	0.471
Communicate with Policy Makers (1 = Never; 4 = Often)	0.008 (0.015)	0.582
Communicate with Reporters (1 = Never; 4 = Often)	-0.001 (0.015)	0.967
Donation Dollar Amount	-0.263 (0.833)	0.752
Amount Donated to Organizations with Political Agenda (in USD)	-0.501 (0.735)	0.496
Democrat (Includes Lean)	$<$0.001 (0.006)	0.943
Republican (Includes Lean)	0.006 (0.005)	0.275
Political Ideology (1=Very Con., 5= Very Lib.)	$<$0.001 (0.016)	0.994

Additional Survey Experimental Results

This subsection describes the two experiments embedded in our survey.

Partisan Cues Survey Experiment

In Experiment 1, we randomly divided the survey sample into three groups. The first group received a politically neutral statement about the 2017 March for Science. The second group received the same message with additional content noting that "many March participants wanted to resist Republican party attacks on science and protest federal science policy's general direction." A third group received a message that framed the perceived attacks as coming from the Trump administration rather than the Republican party. All respondents were then asked to indicate their support or opposition to the March for Science on a 5-point scale from "strongly support" to "strongly oppose."

Support for the March for Science was statistically identical across all three experimental groups, as shown in Table 14. We do not find greater support for the March for Science for respondents given the Trump cue or Republican cue, relative to the neutral message. These null results could plausibly be a function of ceiling effects in political support for the Science March.

Table 14: Experiment 1 Results: March for Science Support as a Function of Exposure to Partisan Cue Conditions

Variable	Estimate	p-value
Trump Cue Treatment	0.120 (0.108)	0.268
Republican Cue Treatment	0.113 (0.108)	0.293
(Intercept: Mean Outcome for Neutral Condition)	4.327 (0.086)	<0.001
N	408	
F-statistic	$F(2, 405) = 0.717$	p-value = 0.489

The outcome is the support for the March for Science, in which 1 means "strongly oppose" and 5 means "strongly support." The heteroskedasticity-robust standard errors are reported in the parentheses after the point estimates.

Domain Expertise Survey Experiment

In a separate survey experiment, we presented respondents with a simple vignette describing the climate advocacy behavior of a hypothetical scholar. We varied both the scholar's discipline (atmospheric chemistry, economics) as well as the nature of the scholar's advocacy (communication about atmospheric climate science, public endorsement of a carbon tax, public endorsement of a carbon tax plus endorsement of political candidates who support carbon taxes).

Figure 2: Experiment 2 Results: Comfort with Political Advocacy as a Function of Advocates' Message and Discipline

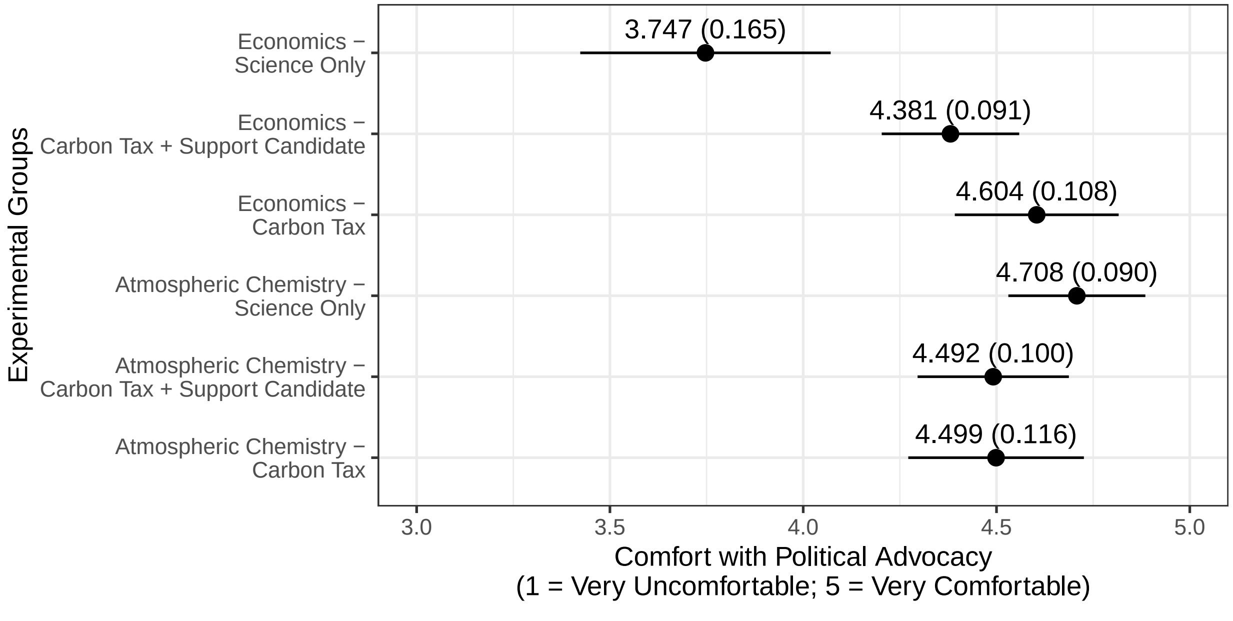

The figure above shows the group mean, standard error, and 95% confidence interval for each experimental group; $N = 408$.

Scientists' comfort with political advocacy does not shift as a function of advocacy content.

Scientists are as comfortable with a peer advocating for a carbon tax and endorsing candidates who advocate for climate policies, as they are with a peer who limits their advocacy to climate science communication. Further, scientists are equally comfortable with scientists and economists making these types of political interventions. The only exception is that scientists are less comfortable with economists intervening in political debates to speak on behalf of climate science.

Text of the survey

The text of the survey appears below.

`NSF apply`: Have you ever applied to the National Science Foundation Graduate Fellowship Program?

- Yes
- No
- I don't remember

Those who replied "No" are taken to a page that states they are not eligible to take the survey.

`edu`: What degrees do you have? Please check all that apply.

- Bachelor's Degree
- Masters Degree
- PhD
- MD
- Law Degree
- Other advanced degree(s)

`identity`: Would you describe yourself as a scientist or social scientist?

- Yes, always
- Yes, sometimes
- No, never

If the subject answered "No, never" to the previous question, they are shown this message:

That's OK! In fact, we want to hear your opinions even more. We hope you'll continue taking our survey. Your opinion is particularly important to us.

`fund`: Within the last five years, have you received any funding for a research project?

- Yes
- No

Those who answered "Yes" to the previous question are shown this following question:

`funder`: Over the past five years, which sources did you receive research funding from? Select as many as apply.

- US federal government
- State government
- Foreign government
- Private foundations or non-profit organizations
- Industry
- Direct support from a university or college
- Scientific professional associations

`group`: Are you a member of the following scientific organizations? Select as many as apply.

- American Association for the Advancement of Science
- National Academy of Sciences
- National Academy of Engineering
- National Academy of Medicine
- Union of Concerned Scientists
- A scientific organization within my discipline
- Other [text box]

`policy1` Which of these statements comes closer to your own view, even if neither is exactly right?

- Scientists should take an active role in public policy debates about issues related to science and technology.
- Scientists should focus on establishing sound scientific facts and stay out of public policy debates.
- I dont know

`policy2`: Which of these statements comes closer to your own view, even if neither is exactly right?

- Scientists should take an active role in public policy debates when they are topic experts on an issue.
- Scientists should focus on establishing sound scientific facts and stay out of public policy debates, even when they are topic experts on an issue.
- I dont know

`fedfund`: Do you think that government funding for scientific research should be increased, kept the same, or decreased?

- Increased
- Kept the same
- Decreased
- I dont know

`nsf1`: Do you think that government funding for the National Science Foundation should be increased, kept the same, or decreased?

- Increased
- Kept the same
- Decreased
- I dont know

`nsf2`: Do you think that government funding for the National Science Foundation Graduate Research Fellowship Program should be increased, kept the same, or decreased?

- Increased

- Kept the same
- Decreased
- I dont know

`comm1`: How often, if ever, do you talk with policymakers about research findings?

- Often
- Occasionally
- Rarely
- Never

`comm2`: How often, if ever, do you talk with reporters about new research findings?

- Often
- Occasionally
- Rarely
- Never

`social1`: How often, if ever, do you post about science on social media?

- Often
- Occasionally
- Rarely
- Never

`social2`: How often, if ever, do you post about political issues on social media?

- Often
- Occasionally
- Rarely
- Never

This is the first of two experiments embedded within the survey. Respondents are randomly assigned to read one of three paragraphs before answering the questions `march1` and `march2`.

Condition 1: The March for Science was a series of rallies and marches held in Washington, DC and over 300 cities across the world on April 22, 2017. The goal of the March was to promote science and to call on politicians and policymakers to enact policies based on scientific evidence.

Condition 2: The March for Science was a series of rallies and marches held in Washington, DC and over 300 cities across the world on April 22, 2017. The goal of the March was to promote science and to call on politicians and policymakers to enact policies based on scientific evidence. Many March participants wanted to resist the Trump Administration's attacks on science and protest federal science policy's general direction.

Condition 3: The March for Science was a series of rallies and marches held in Washington, DC and over 300 cities across the world on April 22, 2017. The goal of the March was to promote science and to call on politicians and policymakers to enact policies based on scientific evidence. Many March participants wanted to resist Republican party attacks on science and protest federal science policy's general direction.

`march1`: Did you support or oppose the March for Science?

- Strongly support
- Somewhat support
- Neither support nor oppose
- Somewhat oppose
- Strongly oppose
- I don't know

`march2`: Did you participate in the March for Science? Select as many as apply.

- I participated in the march in Washington, DC
- I participated in the march in another city
- I donated to the March for Science
- I did not participate in the March for Science

This is the second of two experiments embedded within the survey. Respondents are randomly assigned to read the following before answering the question `comfort`.

`comfort experiment`: Imagine you have a colleague who studies climate change. Their specific expertise is on [economics/climate science]. In recent years, your colleague has become frustrated with the slow pace of climate change policy action in the United States. This colleague has begun to make public statements, speeches, and testimony in support of the US taking more aggressive climate [economics/climate science].

`comfort`: How comfortable are you with the behavior of your colleague?

- Very comfortable
- Somewhat comfortable
- Neither comfortable or uncomfortable
- Somewhat uncomfortable
- Very uncomfortable

`age`: In what year were you born? [Drop-down menu]

`race`: What is your race or origin? Select as many as apply.

- White
- Hispanic, Latino or Spanish origin
- Black or African American
- Asian
- Native American
- Native Hawaiian or Other Pacific Islander
- Other

`employ`: What is your current employment status?

- Working full time now
- Working part time now
- Temporarily laid off
- Unemployed
- Student
- Homemaker

- Retired
- Permanently disabled
- Other

Those who selected “working full time now” or “working part time now” are asked the following question:

`employer`: Which of these best describes your current employer?

- University or college
- Business or industry
- Government
- Non-profit organization
- Other

`field`: What is your primary field or scientific discipline?

- Chemistry
- Computer and Information Sciences & Engineering
- Engineering
- Geoscience
- Life Science
- Materials Research
- Mathematical Science
- Physics & Astronomy
- Psychology
- Social Science
- STEM Education & Learning Research
- I no longer work in any scientific discipline

`citizen`: Which of these statements best describes you?

- I was born in the USA.
- I am an immigrant to the USA and a naturalized citizen.
- I am an immigrant to the USA but not a citizen.

Those who indicated that they are US citizens are asked the following question:

`register` Which of these statements best describes you?

- I am absolutely certain that I am registered to vote in my precinct, election district, or by mail.
- Im not sure if I am registered to vote.
- I am not registered to vote.

`party`: In politics today, do you consider yourself a Republican, Democrat, Independent, or something else?

- Republican
- Democrat
- Independent
- Something else

Those who answered “Independent” or “Something else” in the previous question is asked the following question:

`party`: As of today, do you lean more to the Republican Party or more to the Democratic Party?

- Republican Party
- Democratic Party
- I do not lean to either party

`ideo`: In general, would you describe your political views as...

- Very conservative
- Conservative
- Moderate

- Liberal
- Very Liberal

`amount`: Thank you for completing this survey! You have been entered into a lottery to win one of three $100 cash prizes. If you want, we can donate a portion of your prize to one of the following organizations should you win the prize. Please let us know below how much you would like to donate and which organization you would like to donate.
Optional Donation Amount:

`org`: Preferred Donation Organization:

- American Association for the Advancement of Science
- American Cancer Society
- Union of Concerned Scientists
- The Red Cross
- March for Science
- None

End of the Survey Text:

Thanks for your participation in this survey! Your responses will help us better understand the role of science in American society today. We will be in touch by email if you are selected to win one of the three $100 cash prizes.

We welcome feedback from you. If you have suggestions or comments for us, please write in the textbox below.
